# Prominent transcriptomic changes in *Mycobacterium intracellulare* under acidic and oxidative stress

**DOI:** 10.1186/s12864-024-10292-4

**Published:** 2024-04-17

**Authors:** Hyun-Eui Park, Kyu-Min Kim, Jeong-Ih Shin, Jeong-Gyu Choi, Won-Jun An, Minh Phuong Trinh, Kyeong-Min Kang, Jung-Wan Yoo, Jung-Hyun Byun, Myung Hwan Jung, Kon-Ho Lee, Hyung-Lyun Kang, Seung Cheol Baik, Woo-Kon Lee, Min-Kyoung Shin

**Affiliations:** 1 Department of Microbiology, College of Medicine, Gyeongsang National University, Jinju, 52727 Republic of Korea; 2 Department of Convergence of Medical Science, Gyeongsang National University, Jinju, Republic of Korea; 3 Department of Internal Medicine, Gyeongsang National University Hospital, Jinju, Republic of Korea; 4 Department of Laboratory Medicine, Gyeongsang National University Hospital, Jinju, Republic of Korea

**Keywords:** Mycobacteria, Nontuberculous mycobacteria, *Mycobacterium avium* complex, *Mycobacterium intracellulare*, Stress resistance, RNA-seq, Nitrogen metabolism, Sulfur metabolism

## Abstract

**Background:**

*Mycobacterium avium* complex (MAC), including *Mycobacterium intracellulare* is a member of slow-growing mycobacteria and contributes to a substantial proportion of nontuberculous mycobacterial lung disease in humans affecting immunocompromised and elderly populations. Adaptation of pathogens in hostile environments is crucial in establishing infection and persistence within the host. However, the sophisticated cellular and molecular mechanisms of stress response in *M. intracellulare* still need to be fully explored. We aimed to elucidate the transcriptional response of *M. intracellulare* under acidic and oxidative stress conditions.

**Results:**

At the transcriptome level, 80 genes were shown [FC] ≥ 2.0 and *p* < 0.05 under oxidative stress with 10 mM hydrogen peroxide. Specifically, 77 genes were upregulated, while 3 genes were downregulated. In functional analysis, oxidative stress conditions activate DNA replication, nucleotide excision repair, mismatch repair, homologous recombination, and tuberculosis pathways. Additionally, our results demonstrate that DNA replication and repair system genes, such as *dnaB*, *dinG*, *urvB*, *uvrD2*, and *recA*, are indispensable for resistance to oxidative stress. On the contrary, 878 genes were shown [FC] ≥ 2.0 and *p* < 0.05 under acidic stress with pH 4.5. Among these genes, 339 were upregulated, while 539 were downregulated. Functional analysis highlighted nitrogen and sulfur metabolism pathways as the primary responses to acidic stress. Our findings provide evidence of the critical role played by nitrogen and sulfur metabolism genes in the response to acidic stress, including *narGHIJ*, *nirBD*, *narU*, *narK3*, *cysND*, *cysC*, *cysH*, *ferredoxin* 1 and 2, and *formate dehydrogenase*.

**Conclusion:**

Our results suggest the activation of several pathways potentially critical for the survival of *M. intracellulare* under a hostile microenvironment within the host. This study indicates the importance of stress responses in *M. intracellulare* infection and identifies promising therapeutic targets.

**Supplementary Information:**

The online version contains supplementary material available at 10.1186/s12864-024-10292-4.

## Background

Non-tuberculous mycobacteria (NTM) are environmental mycobacteria other than *M. tuberculosis* complex and *M. leprae*, comprising more than 180 species [1]. Most NTM are non-pathogenic to humans, but some species cause infection in patients with immunocompromised conditions and structural lung disease [2–4]. The incidence and prevalence of NTM infections have steadily increased over the last several decades and emerged as a significant global public health concern [5–8]. *Mycobacterium avium* complex (MAC), including *Mycobacterium avium* and *Mycobacterium intracellulare*, is the most common causative agent of NTM-lung disease in the world and affects susceptible populations with certain risk factors such as bronchiectasis, chronic obstructive pulmonary disease, autoimmune disease, and aging [9].

Mycobacteria can adapt to stress conditions, including oxidative stress, nutrient starvation, pH change, and temperature, which elucidate its evolutional adaptation by a refined network of molecular mechanisms [10–13]. The production of reactive oxygen species (ROS) and the acidification of phagosomes are the critical mechanisms that macrophages employ to kill internalized pathogens during infections [14–17]. Consequently, mycobacteria have evolved defense mechanisms to protect themselves against host-induced stress [18–20]. Under oxidative stress conditions, two global transcription factors, OxyR and SoxRS, regulate the stress response in many bacterial species [21]. OxyR responds to peroxide stress, while SoxRS responds to superoxide stress [14]. However, pathogenic mycobacteria such as *M. tuberculosis* and *M. leprae* lack the general antioxidant mechanisms employed by most other intracellular bacterial pathogens [22]. Previous studies have reported that pathogenic mycobacteria exhibit multiple mutations within the oxyR gene, resulting in a dysfunctional protein [22]. Although *M. tuberculosis* lacks the functional *oxyR* gene, several other genes associated with the oxidative stress response, such as *ahpC*, *katG*, and *furA*, remain functional [23]. The presence of alternative detoxification pathways has the potential to act as a compensatory mechanism.

For example, Lu et al. investigated the proteomic profiling of *Mycobacterium tuberculosis* (Mtb) under oxidative stress, focusing on total cysteine thiols modification and S-sulfenylation modification [10]. They identified the differential expression of numerous proteins at total cysteine modification and S-sulfenylation modification levels under hydrogen peroxide exposure [10]. These cysteine-modified proteins were associated with oxidation–reduction, fatty acid synthesis, cell wall remodeling, and protein repair [10]. Recently, Yimcharoen et al. investigated the transcriptional response of drug susceptible and resistant Mtb strains after isoniazid exposure under stress conditions that mimic the host environment [13]. The expression of stress-response genes such as *hspX*, *tgs1*, *icl1*, and *sigE* and lipoarabinomannan (LAM) synthesis associated genes including *pimB*, *mptA*, *mptC*, *dprE1*, *dprE2*, and *embC* were highly differentiated between the drug-susceptible and resistant strains [13]. These findings suggest the role of stress response and LAM synthesis-associated genes may be pivotal for the adaptation and persistence of Mtb within the host. Similarly, Martini et al. showed that small non-coding RNA MTS1338 promoted distinct expression profiles for stress response in Mtb under macrophage-like stress conditions, suggesting stress-triggered small non-coding RNA enhances bacterial survival within the host by inducing global transcriptional changes [11].

Despite contradictory results, exposure to NTM from the environmental niche, such as soil and water, has been proposed as a source of infection. Tzou et al. demonstrated that the isolation rate of NTM from shower aerosols is higher in the NTM patient's homes than in control homes based on Washington and Oregon residents compared with age, sex, and geography-matched controls [24]. Furthermore, Reed et al. revealed that prolonged soil exposure is associated with *M. avium* complex exposure in a cross-sectional study in Florida by population-based random household survey [25]. Similarly, clinically dominant NTM species are present in patient’s potting soil [26]. Interestingly, NTM isolates from potting soil and patients showed similar restriction enzyme digestion patterns by PFGE [26]. On the contrary, Choi et al. reported that NTM species recovered from patients did not match in the showerheads [27]. NTM isolates from the environment and patients share similar characteristics, and elucidating the stress response of NTM isolates recovered from environmental sources may reveal the survival strategy within the host for successful infection. Therefore, we performed comprehensive transcriptional profiling of *Mycobacterium intracellulare* isolate recovered from soil exposed to oxidative and acidic stress conditions. We also identified differential gene groups and pathways in response to stress conditions. Our findings provide a better understanding of the molecular mechanisms for adaptation to the host-induced stress conditions in *Mycobacterium intracellulare* and possibly other MAC species.

## Material and methods

### Bacterial strains and growth conditions

*Mycobacteria intracellulare* S1-36 strain, that isolated from soil samples of South Korea in 2019 was used in this study. *M. intracellulare* S1-36 strain harbors 5.4 Mbp genome and showed similar genetic feature with *M. intracellulare* ATCC13950 strain through the whole genome sequencing analysis [28]. *M. intracellulare* S1-36 strain were cultured in Middlebrook 7H9 broth (MB7H9; Becton, Dickinson and Company) supplemented with 0.5% glycerol and 10% ODAC at 37 ℃ and 250 rpm.

### In vitro stress conditions

The *M. intracellulare* S1-36 strain was inoculated onto 7H10 agar (Becton, Dickinson and Company), which was supplemented with 10% OADC, and then incubated at 37 °C for 3 weeks. A single colony of *M. intracellulare* was subsequently transferred to 10 mL of MB7H9 medium and incubated at 37 °C with shaking at 250 rpm until it reached an OD_600_ of 0.5. The mid-log phase *M. intracellulare* cultures were washed with 1 × PBS for 3 times and then transferred into 10 mL of Sauton’s media with either a final concentration of 10 mM hydrogen peroxide for oxidative stress or acidic Sauton’s media (pH 4.5) for acidic stress. Samples were incubated at 37 °C with shaking at 250 rpm for 16 h. Untreated *M. intracellulare* cultures were used as a control. All samples were prepared in biologically independent three biological replicates.

### RNA extraction

We extracted total RNA from a culture of *M. intracellulare* using the RNeasy Plus Kits (Qiagen), according to the manufacturer's instructions with slight adjustments. In summary, following 16 h of exposure to stress conditions, the samples were centrifuged at 4000 rpm at 20 °C for 20 min. Subsequently, we subjected them to three washes with 1 × PBS and then resuspended them in 2 mL of RNAprotect Bacteria Reagent from Qiagen. To stabilize the transcriptional profile, we incubated the samples at room temperature for 5 min. Afterward, we centrifuged the samples at 4000 rpm at 20 °C for 20 min and resuspended them in 1 mL of RLT buffer containing 300 μL of 0.1 mm zirconia beads (BioSpec). The samples, inclusive of mycobacterial cells, underwent lysis at 4500 rpm for 45 s, repeated three times using the Precellys 24 homogenizer (Bertin Technologies). Following lysis, we centrifuged the samples at 13,000 rpm for 5 min, and subsequently transferred 700 μL of the supernatant to a 2 mL tube. We proceeded with the remaining RNA extraction steps as per the established protocol.

### Library preparation and sequencing

The purified RNA samples were dispatched to Macrogen (Seoul, South Korea). We assessed the integrity of all RNA samples using an Agilent 2100 Bioanalyzer (Agilent Technologies, Waldbronn, Germany). Notably, all RNA samples displayed RNA integrity numbers greater than or equal to 8.6. We performed rRNA removal using the NEBNext rRNA Depletion kit and constructed sequencing libraries using the TruSeq Stranded Total RNA Library Prep Gold Kit by Illumina. The generated libraries were subjected to gel purification and were validated by evaluating size, purity, and concentration using the Agilent Bioanalyzer. For library quantification, we followed the qPCR Quantification Protocol Guide utilizing KAPA Library Quantification kits for Illumina Sequencing platforms. Subsequently, we conducted sequencing on an Illumina NextSeq, generating paired-end reads (2 × 101 bp). Image decomposition and quality value calculations were carried out using the modules within the Illumina pipeline. Macrogen conducted all procedures related to next-generation sequencing analysis.

### Sequencing data analysis

FastQC was employed to assess the quality of sequences, and only sequences with a Phred quality score ≥ 30 were retained for further analysis. The sequencing reads were then aligned to the reference genome *M. intracellulare* ATCC13950 [29] using the Bowtie aligner. Following read mapping, read counts for each gene in each sample were extracted based on the gene annotations specific to the corresponding species using the HTSeq program. Differential expression analysis was carried out using DESeq, applying the criteria of |fold change|≥ 2 and a raw *p*-value from the nbinomWaldTest < 0.05.

### Pathway enrichment analysis

We conducted pathway enrichment analysis, which identifies biologically relevant pathways by assessing the overlap between genes of interest and specific gene sets or pathways from the Kyoto Encyclopedia of Genes and Genomes (KEGG) pathway database [30]. We employed Fisher’s exact test to identify statistically significant KEGG pathways, and corrected *p*-values were calculated using the Benjamini and Hochberg false discovery rate algorithm.

### RNA-seq data validation

To validate the RNA-seq data, we selected nine differentially expressed genes (DEGs) for quantitative real-time PCR (qRT-PCR). The synthesis of cDNA was performed following the manufacturer’s instructions. In brief, 16 µl of samples containing 500 ng of RNA were incubated at 70 ℃ for 5 min to dissociate secondary structure within the RNA sample and chill the tube immediately on ice. Subsequently, we added 4 µl of reverse transcription master mix (ELPIS biotech, Korea) and incubate for 60 min at 37 ℃. Finally, the samples were incubated for 5 min at 94 ℃ to stop reaction. The cDNA samples were diluted for 1:10 with nuclease free water and used for further analysis. We performed qRT-PCR using a SsoFast Eva Green Supermix (Bio-Rad) and Rotor-Gene Q real-time PCR cycler (Qiagen). Amplification was performed for 40 cycles at 95 °C for 10 s, followed by 30 s at 62 °C with fluorescence detected during the extension step. The relative gene expression level was calculated by the 2^−ΔΔCt^ method using beta subunit of RNA polymerase (*rpoB*) as a reference gene. The sequences of primers for qPCR experiment used in present study are listed in Table 1.

**Table 1:** Oligonucleotide sequences of primers used in qPCR validation

Gene name	Forward primer (5’ to 3’)	Reverse primer (5’ to 3’)
*rpoB*	ACCTCGGTGGTCAGGTAGTA	GGAAGGCAAGGCAATTCAGC
*narH*	CAACCACAAGACGGGCAAAG	TCGGATCGTTAGGGTCCAGT
*narJ*	CGCCACCATGTATCTGACGTA	GTGGCACGGTCAAGGTAAAC
*narI*	GAAGATGAGCGACCACGTCT	AACCACAAACCGACCGTGTA
*narU*	CGAGGAAGAACGCGATGTAGA	TACCGGATGATTTCGCGGAT
*groEL1*	GCTCTCCTTGCGTTCCTTGA	GAGGATCTGGCGATCGTGAC
*groEL2*	CTTCGCTGATGACCTGACCA	AAGGGCTACATCTCGGGCTA
*dinB*	GTTGGGCGACATGGCATTAC	CCCGATCAGGAGTTGACGTT
*mmpS*	GATGAACGGTTTGATGGCGT	GGGACCGCGAATCTGAACTA
*groES*	TTACTTGGAGACGACAGCCA	GAAGGCGACACCGTCATCTA

## Results and discussion

In the present study, we investigated the transcriptomic changes of environmental *M. intracellulare* isolate under acidic and oxidative stress conditions that mimic the stress induced by the host environment. Previous studies have investigated pathogenic mycobacteria's transcriptional and proteomic profiles under various stressors, such as hypoxia, nutrient deprivation, changes in pH, and oxidative stress [31–39]. However, these prior investigations predominantly centered on *M. tuberculosis* rather than the MAC. Consequently, our understanding of the transcriptome of MAC, particularly in the context of *M. intracellulare* under host-induced stress conditions, still needs to be improved. This study represents the first effort to elucidate the specific transcripts expressed by *M. intracellulare* when exposed to acidic and oxidative stress conditions.

Macrophages are the first-line defense in the host immune response against intracellular bacteria, including *M. tuberculosis* and MAC [40, 41]. Once encountered, macrophages recognize and engulf bacteria via interaction between pathogen-associated molecular patterns and pattern recognition receptors [42]. Upon phagocytosis, various antimicrobial mechanisms are activated within the macrophages. The maturation of phagosomes into phagolysosomes induces its acidification modulated by the Abl tyrosine kinase and vacuolar-type ATPase [43]. This process is required to activate degradative enzymes, including hydrolase and cathepsins, showing optimal activity at low pH. [43]. Also, activated macrophages produce various antimicrobial effector molecules, such as antimicrobial peptides, lipid mediators, and oxygen and nitrogen radicals, to sterilize engulfed pathogens [44]. However, pathogenic mycobacteria can endure host-induced stress condition and survive within the macrophages by arresting the maturation of phagosome and lysosomal fusion [44].

### Summary of RNA-sequencing data

The RNA samples' concentration ranged from 43.3 to 102.3 ng/µl. Also, all RNA samples showed an RNA integrity number score of ≥ 8.6 and rRNA ratio of > 1.0. RNA-seq library preparation was conducted with extracted total RNA from *M. intracellulare* under oxidative and acidic stress conditions. RNA-seq produced 32.3 to 36.8 million total reads per library. FastQC analyzed the quality of sequences, and sequences with a phred quality score ≥ 30 were used for further analysis. After eliminating the reads with low-quality bases and adapter sequences by the Trimmomatic program, the remaining sequences were 31.6 to 36.1 million reads per library (Table 2). Bowtie aligner mapped trimmed readds with reference genome *M. intracellulare* ATCC13950 (ASM27712v1). The processed reads ranged from 31.5 to 36 million reads per library and the rate of mapped reads with its reference genome ranged from 86.1 to 88.1% (Table 2). Collectively, a high-quality cDNA library was constructed and used for further RNA-seq data analysis.

**Table 2:** Summary statistics of sequence quality and alignment information of analysis

Sample ID	Total reads	Processed reads	Q20 (%)	Q30 (%)	GC (%)	Mapping rate (%)
S1-36_Con1	32,690,722	32,690,722	98.92	96.35	63.17	87.43
S1-36_Con2	36,009,040	36,009,040	98.83	96.05	63.41	87.23
S1-36_Con3	35,806,154	35,806,154	98.84	96.11	62.75	86.80
S1-36_A1	31,551,876	31,551,876	98.95	96.43	63.29	86.61
S1-36_A2	35,740,226	35,740,226	98.87	96.18	63.09	86.92
S1-36_A3	35,913,930	35,913,930	98.85	96.13	63.19	87.03
S1-36_O1	35,884,918	35,884,918	98.88	96.20	62.40	88.10
S1-36_O2	35,358,014	35,358,014	98.87	96.17	62.84	87.73
S1-36_O3	33,547,994	33,547,994	98.94	96.40	63.24	86.26

### Multidimensional scaling

**Fig. 1:**
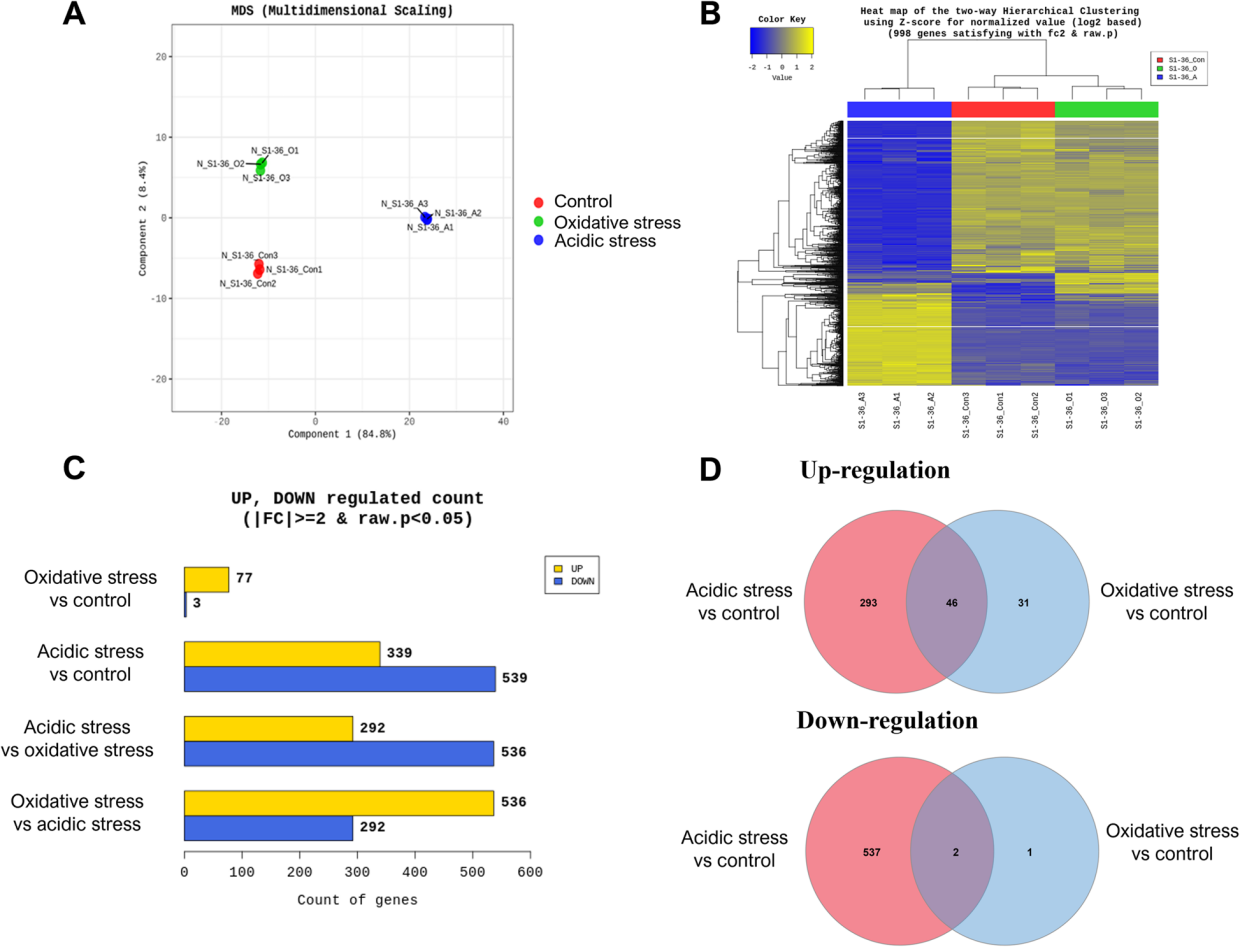
Transcriptional profiling of *M. intracellulare* under oxidative and acidic stress conditions. The transcript data samples were analyzed from *M. intracellulare* cultures consisting of three groups as follows: Con (control): *M. intracellulare* cultures in pH 7.0 Sauton’s media, O (oxidative stress): *M. intracellulare* cultures in pH 7.0 Sauton’s media treated with 10 mM hydrogen oxide, A (acidic stress): *M. intracellulare* cultures in pH 4.5 Sauton’s media. Three biological replicates were used for analysis. **A** Multidimensional scaling plots of samples from transcript data samples. Three biological replicates have similar expression patterns in all experimental groups. **B** Cluster heatmap of differentially expressed genes (|fold change|≥ 2 and raw *p* < 0.05) in nine transcript data samples. **C** Numbers of differentially expressed genes in RNA-seq. The differentially expressed genes were defined as |fold change|≥ 2 and raw *p* < 0.05. **D** Venn diagram showing the shared genes that were significantly up- or down-regulated in *M. intracellulare* culture under oxidative and acidic stress conditions. Filter was set at |fold change|≥ 2 and raw *p* < 0.05

Multidimensional scaling analysis plot showed the variability of the samples between control, acidic stress, and oxidative stress groups (Fig. 1A). The results revealed a distinct clustering of the three biological replicates of RNA samples belonging to each group. These data suggest that all the variations caused by biological replicates have been normalized, and each group was separately clustered.

### Differentially expressed genes (DEGs) in M. intracellulare under acidic and oxidative stress

We conducted transcriptomic analysis under oxidative and acidic stress conditions. The DESeq2 analysis showed that 998 differentially expressed genes (DEGs) were significantly expressed *in M. intracellulare* exposed to acidic and oxidative stress conditions compared to the control group, filtered by |fold change|≥ 2.0 and raw *p*-value < 0.05 (Fig. 1B). 878 DEGs were observed in the acidic stress group. Among them, 339 DEGs were significantly upregulated, while 539 DEGs were significantly downregulated (Fig. 1C). On the contrary, relatively small numbers of DEGs were observed in the oxidative stress group. In total, 80 DEGs were expressed in the oxidative stress group compared to the control group (Fig. 1C). Among them, 77 DEGs were significantly upregulated, while three DEGs were significantly downregulated in the oxidative stress group compared to the control group (Fig. 1C).

**Fig. 2:**
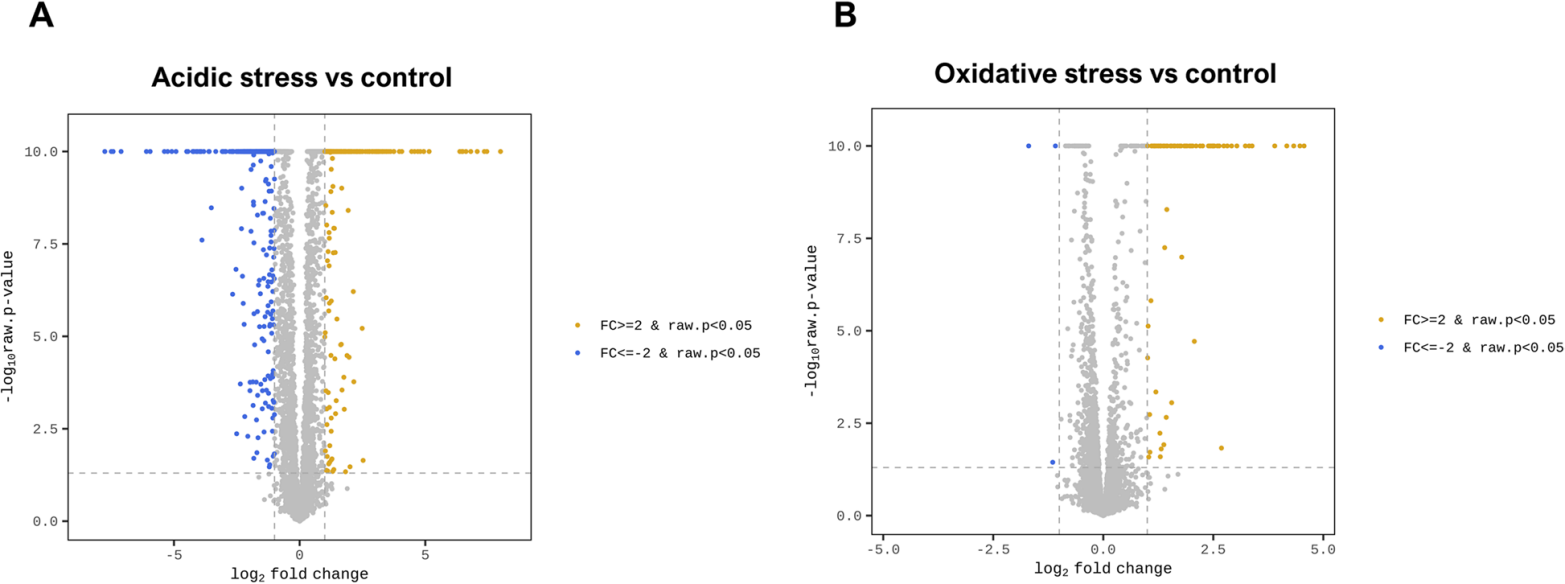
Volcano plots of *M. intracellulare* transcriptome under oxidative and acidic stress conditions. **A** Volcano plots comparing transcriptional levels between acidic stress group compared to control group. Yellow dots indicate an expression level change of fold change ≥ 2 and raw *p* < 0.05. Blue dots indicate an expression level change of fold change ≤ -2 and raw *p* < 0.05. Gray dots indicate no significant expression level change. **B** Volcano plots comparing transcriptional levels between oxidative stress group compared to control group. Yellow dots indicate an expression level change of fold change ≥ 2 and raw *p* < 0.05. Blue dots indicate an expression level change of fold change ≤ -2 and raw *p* < 0.05. Gray dots indicate no significant expression level change

A Venn diagram showed 48 DEGs were overlapped between two groups (Fig. 1D). Among them, 46 DEGs were upregulated, while two DEGs were downregulated (Fig. 1D). Also, 293 and 537 DEGs were uniquely upregulated and downregulated in acidic stress group, respectively (Fig. 1D). Furthermore, 31 and one DEGs were uniquely upregulated and downregulated in oxidative stress group, respectively (Fig. 1D). Subsequently, we produced volcano plots analyzing the fold changes in expression with the corresponding raw *p*-values (Fig. 2). Volcano plot showed differentially expressed transcripts in acidic and oxidative stress conditions compared to the untreated control (Fig. 2A and B). Classification of these 48 DEGs by their functional categories based on Mycobrowser showed that the most relevant functional categories were “information pathways” (12.5%), “insertion seqs and phages” (10.4%), “intermediary metabolism and respiration” (6.3%), and “virulence, detoxification, adaptation” (6.2%). Description of the overlapped transcripts between acidic and oxidative stress conditions were listed in Table 3.

**Table 3:** Description of overlapped DEGs between acidic and oxidative stress conditions

Gene ID	Function	Functional categories	Fold change in acidic stress condition	Fold change in oxidative stress condition
OCU_t00110	tRNA-Thr	Stable RNAs	2.16	2.49
OCU_00590	hypothetical protein	Unknown	2.30	2.05
OCU_00810	MmpS family protein	Cell wall and cell processes	5.61	2.46
OCU_01820	DNA polymerase IV	Information pathways	2.23	9.38
OCU_02460	hypothetical protein	Insertion seqs and phages	3.90	20.09
OCU_03240	hypothetical protein	Conserved hypotheticals	4.05	5.42
OCU_04530	hypothetical protein	Insertion seqs and phages	4.49	18.01
OCU_05560	hypothetical protein	Conserved hypotheticals	3.82	2.29
OCU_06830	zinc transporter Slc39a7	Cell wall and cell processes	-2.95	-2.13
OCU_06840	hypothetical protein	Regulatory proteins	-3.42	-3.24
OCU_08780	hypothetical protein	Conserved hypotheticals	4.88	14.89
OCU_08910	hypothetical protein	Unknown	2.19	3.65
OCU_09680	hypothetical protein	Unknown	3.39	2.70
OCU_09740	putative regulatory protein, FmdB family protein	Unknown	3.44	2.16
OCU_10570	hypothetical protein	Unknown	2.40	2.63
OCU_11420	glyoxalase family protein	Unknown	2.73	5.75
OCU_11450	hypothetical protein	Unknown	2.64	3.44
OCU_18370	hypothetical protein	Unknown	5.76	6.43
OCU_21410	TetR family transcriptional regulator	Regulatory proteins	3.22	2.94
OCU_21470	fdxC_1	Intermediary metabolism and respiration	2.93	4.78
OCU_21490	secreted protein	Intermediary metabolism and respiration	9.86	4.60
OCU_21500	nirA_1	Intermediary metabolism and respiration	13.48	5.64
OCU_22710	hypothetical protein	Unknown	3.58	3.90
OCU_26160	hypothetical protein	Conserved hypotheticals	2.12	6.04
OCU_27960	hypothetical protein	Conserved hypotheticals	2.34	5.24
OCU_29330	hypothetical protein	Conserved hypotheticals	3.10	5.68
OCU_29740	excinuclease ABC subunit B	Information pathways	3.04	2.71
OCU_32850	13e12 repeat-containing protein	Insertion seqs and phages	4.06	5.55
OCU_34460	LysM domain-containing protein	Unknown	2.08	3.40
OCU_36850	hypothetical protein	Conserved hypotheticals	3.48	4.09
OCU_38160	hypothetical protein	Conserved hypotheticals	5.63	9.95
OCU_39980	hypothetical protein	Information pathways	4.06	5.36
OCU_39990	helicase, UvrD/Rep family protein	Information pathways	4.19	7.55
OCU_41250	DEAD/DEAH box helicase	Information pathways	2.58	3.04
OCU_41260	hypothetical protein	Unknown	2.27	2.38
OCU_41760	hypothetical protein	Insertion seqs and phages	3.20	3.34
OCU_41930	error-prone DNA polymerase	Information pathways	3.61	2.25
OCU_42030	hypothetical protein	Conserved hypotheticals	3.17	22.14
OCU_42040	hypothetical protein	Conserved hypotheticals	3.92	23.63
OCU_42300	chaperonin GroEL	Virulence, detoxification, adaptation	3.48	3.07
OCU_42310	chaperone GroES	Virulence, detoxification, adaptation	4.00	3.23
OCU_42900	hypothetical protein	Unknown	4.40	2.08
OCU_43250	13e12 repeat-containing protein	Insertion seqs and phages	3.73	10.42
OCU_44140	hypothetical protein	Conserved hypotheticals	3.94	5.64
OCU_45770	chaperonin GroEL	Virulence, detoxification, adaptation	4.78	3.73
OCU_46150	hypothetical protein	Conserved hypotheticals	3.83	8.20
OCU_47420	hypothetical protein	Unknown	3.70	2.44
OCU_49070	hypothetical protein	Unknown	3.19	6.68

We observed transcriptional changes in general stress in the present study, indicating genes differentially expressed under both stress conditions. A total of 48 genes were differentially expressed under both stress conditions. Among 48 DEGs, 46 genes were upregulated, whereas two were downregulated. The upregulated genes are classified into various categories, such as [intermediary metabolism and respiration], [information pathways], [virulence, detoxification, adaptation], [insertion seqs and phages], [cell wall and cell processes], and [regulatory proteins]. Also, several genes were classified into [unknown] and [conserved hypotheticals]. The genes encoding molecular chaperones such as *groEL1*, *groEL2*, and *groES* were upregulated under both stress conditions. Mycobacteria have two types of molecular chaperones, such as GroEL1 and GroEL2, and a single *groES* gene, which is combined with groEL1 at the transcriptional level [45]. Previous studies demonstrated that GroEL1 and GroEL2 proteins are upregulated under heat shock, thiol-specific oxidative stress, and macrophage infection [46–48]. Furthermore, GroEL1 and GroEL2 induce cytokine production in human PBMC, suggesting that these proteins are essential virulence factors in mycobacterial infection [49]. Also, the mycobacterial GroEL1 protein is involved in biofilm formation. Ojha et al. showed that GroEL1 promotes the formation of mature biofilms by modulating mycolic acid biosynthesis [50]. *M. smegmatis groEL1* deletion mutant showed normal planktonic growth but could not produce biofilm [50]. Besides GroEL proteins, GroES proteins provoke immune responses, such as T cell proliferation and immunoglobulin response in tuberculosis and leprosy [51–53]. In *M. avium* complex, the role of molecular chaperones, such as GroEL1, GroEL2, and GroES in pathogenesis remains undiscovered. The construction of genetic mutants to elucidate the role of general stress response genes is needed in further investigation.

### RNA-seq data validation

**Fig. 3:**
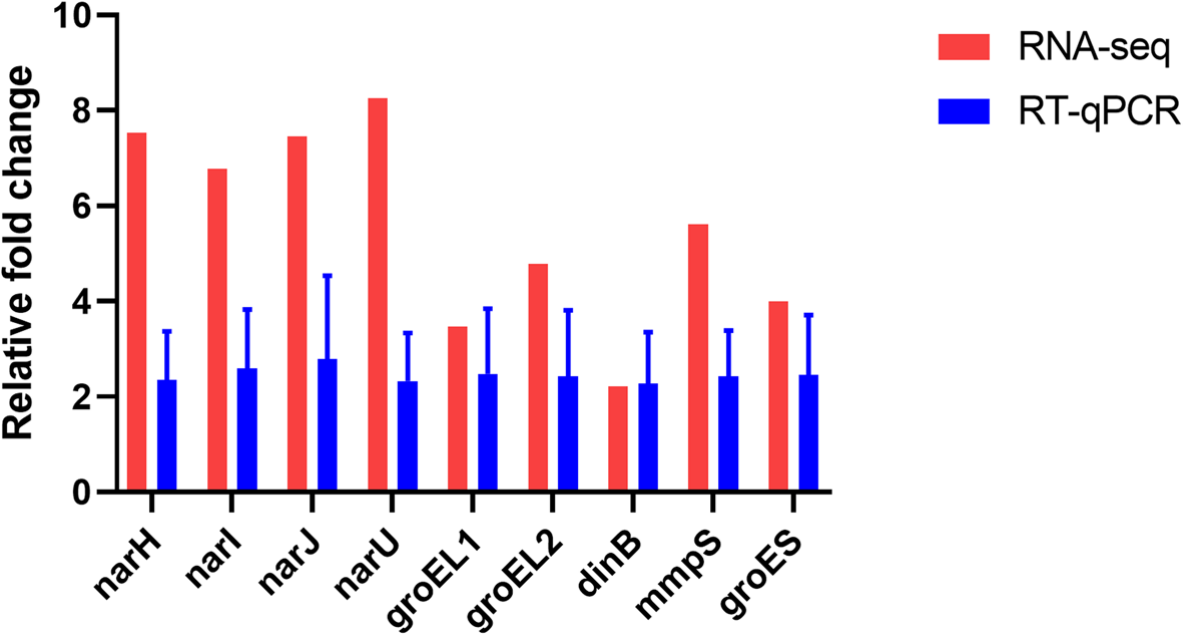
Validation of RNA-seq data by quantitative RT-PCR. The relative gene expression level of nine DEGs in acidic stress condition was normalized to the *rpoB* expression level relative to control group by the 2^−ΔΔCT^ method

We tested nine DEGs' expression levels by RT-qPCR to validate the RNA-seq data. The mRNA levels of nine DEGs such as *narH*, *narI*, *narJ*, *narU*, *groEL1*, *groEL2*, *dinB*, *mmpS*, and *groES*, were upregulated in the acidic stress condition, consistent with the RNA-seq data. However, their expression levels were varied among the different DEGs. For instance, expression level of respiratory nitrate reductase complex including *narH*, *narI*, *narJ*, and *narU* was increased in acidic stress condition, we only observed 2.32-to-2.78-fold upregulation in the RT-qPCR result (Fig. 3). Similarly, the mRNA levels of molecular chaperones such as *groEL1*, *groEL2*, and *groES* were upregulated for 3.47-to-fourfold in acidic stress condition, we only detected 2.42-to-2.47-fold upregulation in the RT-qPCR analysis (Fig. 3). The difference in sensitivity and dynamic range between RNA-seq and qRT-PCR could explain the difference of fold change between two methods.

### KEGG enrichment analysis

**Fig. 4:**
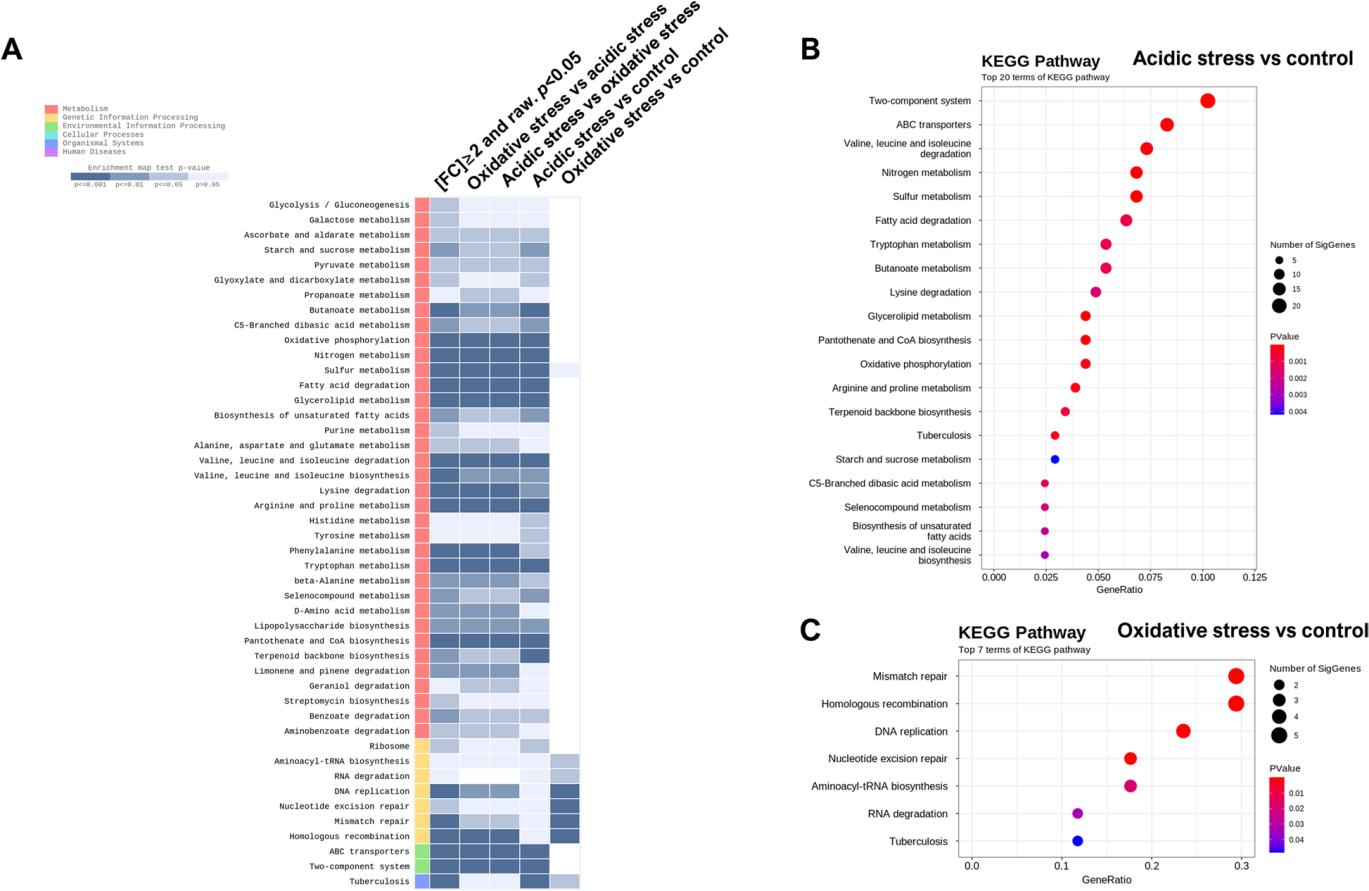
KEGG enrichment analysis of *M. intracellulare* transcriptome under oxidative and acidic stress conditions. **A** KEGG enrichment pathways that are differentially expressed in the entire experimental groups filtered by *p* < 0.05. **B** Top 20 KEGG enrichment pathways from acidic stress group compared to control group. **C** Top 7 KEGG enrichment pathways from oxidative stress group compared to control group. Size of circle indicates the number of significant genes and color of circle indicates *p* value as presented in figure

In this work, we performed KEGG enrichment analysis to provide a deeper insight into the biological mechanisms of up and downregulated DEGs. The result of KEGG enrichment analysis showed that target genes were significantly enriched in pathways belonging to metabolism (36 pathways, 78.2%), genetic information processing (seven pathways, 15.2%), environmental information processing (two pathways, 4.3%), and organismal systems (one pathway, 2.2%) (Fig. 3A). In detail, acidic stress condition activated several pathways such as two-component system, ABC transporters, valine, leucine, and isoleucine degradation, nitrogen metabolism, sulfur metabolism, fatty acid degradation, tryptophan metabolism, butanoate metabolism, lysine degradation, glycerolipid metabolism, pantothenate and CoA biosynthesis, oxidative phosphorylation, arginine and proline metabolism, terpenoid backbone biosynthesis, tuberculosis, starch, and sucrose metabolism, C5-branched dibasic acid metabolism, selenocompound metabolism, biosynthesis of unsaturated fatty acids, and valine, leucine, and isoleucine biosynthesis (Fig. 4B). Furthermore, seven pathways including mismatch repair, homologous recombination, DNA replication, nucleotide excision repair, aminoacyl-tRNA biosynthesis, RNA degradation, and tuberculosis were activated under oxidative stress condition (Fig. 4C). The enriched pathways contain different number of DEGs ranging from 2 to 20. Heatmaps of DEGs belong to enriched pathways were presented in supplementary Fig. 1 and 2.

Next, we selected the key genes potentially involved in the adaptation under acidic and oxidative stress conditions from the top 20 terms of KEGG pathways. A total of 28 and 13 genes were selected from acidic and oxidative stress conditions, respectively (Table 4). Under acidic stress conditions, most of selected DEGs were associated with nitrogen and sulfur metabolism. Also, several genes that involved in potassium transport, membrane stress, and heat shock stress (Table 4). On the contrary, oxidative stress conditions induces the gene expression profiles associated with DNA replication, nucleotide excision repair processes, and liporarabinomannan biosynthesis (Table 4). Overall, these findings indicate that the stress-responsive differentially expressed genes (DEGs) in *M. intracellulare* play a key role in diverse metabolic processes, including nitrogen and sulfur metabolism during acidic stress, as well as involvement in DNA repair, cell wall maintenance, and remodeling when facing oxidative stress.

**Table 4:** Description of key DEGs of M. *intracellulare* under acidic and oxidative stress conditions

Gene ID	Gene name	Functional categories	Fold change in acidic stress condition	Fold change in oxidative stress condition
OCU_47950	narU	Nitrogen metabolism	8.25	-1.04
OCU_48030	narK3		8.70	1.03
OCU_12100	narG		8.85	1.01
OCU_12110	narH		7.53	1.07
OCU_12130	narI		6.77	1.09
OCU_48070	nirB		30.68	-1.10
OCU_48080	nirD		30.66	-1.13
OCU_01750	gltB		2.17	-1.05
OCU_01760	gltD		2.01	-1.04
OCU_07280	narL		-2.87	-1.22
OCU_12120	narJ		7.46	1.12
OCU_10620	kdpA	Potassium limitation	3.12	1.12
OCU_10610	kdpB		5.28	1.24
OCU_10600	kdpC		3.91	1.00
OCU_09670	mprB	Membrane stress	2.41	1.05
OCU_09660	mprA		3.17	1.03
OCU_09690	pepD		2.55	-1.04
OCU_10630	trcS	Heat shock, membrane stress	-10.11	1.14
OCU_10640	trcR		-4.10	1.06
OCU_36170	glnD	Glutamate metabolism	2.12	-1.00
OCU_36180	glnB		2.16	-1.08
OCU_15790	cysN	Sulfur metabolism	2.14	-1.06
OCU_15800	cysD		2.30	-1.03
OCU_18650	cysH		2.27	-1.05
OCU_18660	ferredoxin 2		2.45	-1.03
OCU_21500	ferredoxin		13.48	5.64
OCU_20490	formate dehydrogenase		111.19	1.01
OCU_21100	cysQ		2.32	-1.05
OCU_00740	dnaB	DNA replication	-1.07	2.62
OCU_03510	dinG		-1.60	2.48
OCU_22700	Conserved hypothetical protein		1.17	4.32
OCU_04360	Possible DNA polymerase		1.42	3.09
OCU_41260	nei2 (endonuclease)	DNA repair	2.26	2.38
OCU_12600	DNA-3-methyladenine glycosylase I		1.56	2.21
OCU_29740	uvrB		3.03	2.71
OCU_39940	uvrD2		1.54	2.22
OCU_39980	Probable ATP-dependent DNA helicase		4.06	5.36
OCU_34590	recA		1.24	2.28
OCU_33030	ruvC		-1.38	2.25
OCU_42300	groEL1	Heat shock response	3.47	3.07
OCU_45770	groEL2	Lipoarabinomannan biosynthesis	4.78	3.73

**Fig. 5:**
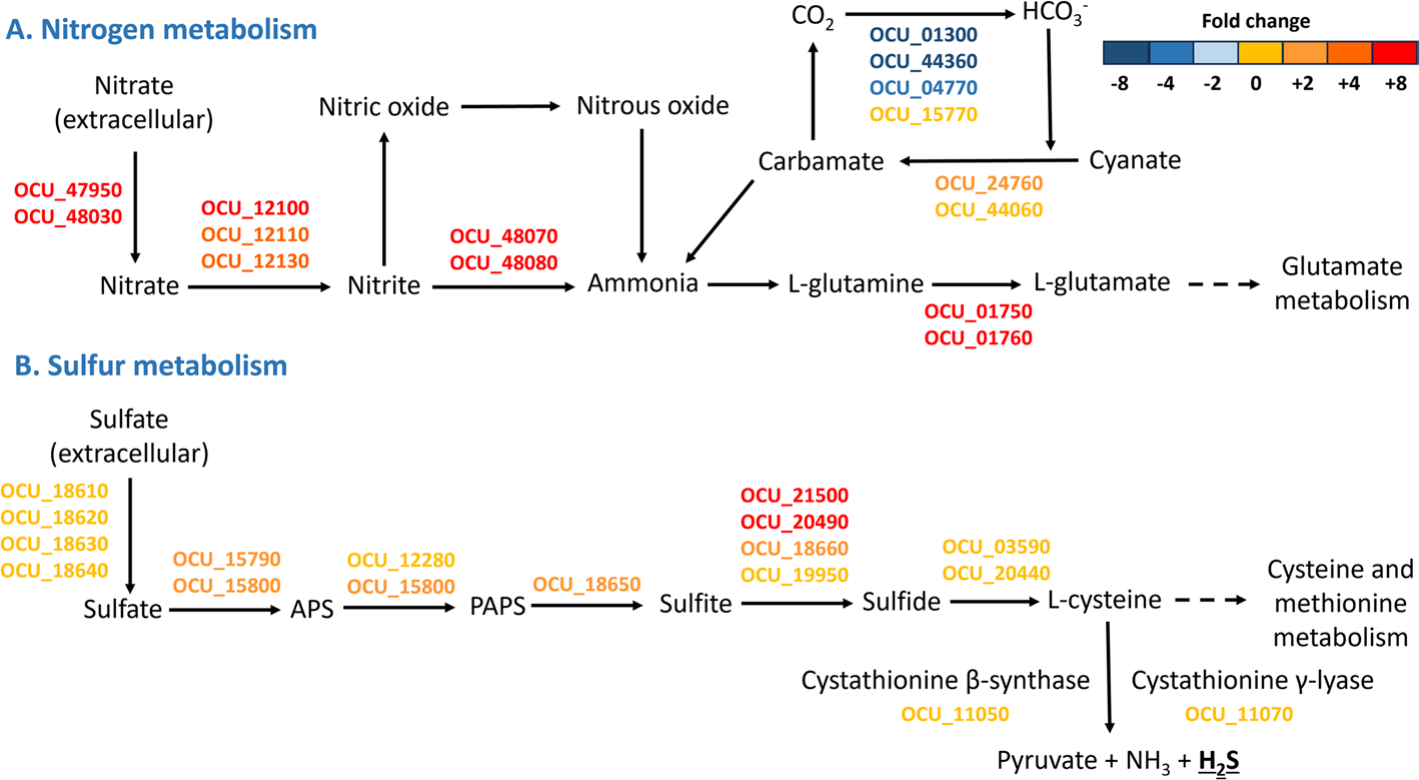
Transcriptomic pathways in *M. intracellulare* that exhibit differential expression in response to acidic stress. **A** The nitrogen metabolism pathways in *M. intracellulare* are presented here and show differential expression under acidic stress. We have depicted gene expression associated with nitrogen metabolism, with the colors of gene IDs indicating their expression levels as described in the figure. The gene expression levels of differentially expressed genes identified under acidic stress were compared with the corresponding transcripts detected under untreated control growth conditions. **B** The sulfur metabolism pathways in *M. intracellulare* are presented here and show differential expression under acidic stress. We have depicted gene expression associated with nitrogen metabolism, with the colors of gene IDs indicating their expression levels as described in the figure. The gene expression levels of differentially expressed genes identified under acidic stress were compared with the corresponding transcripts detected under untreated control growth conditions

We observed a clear distinct activated pathways between the acidic and oxidative stress conditions. Specifically, under acidic stress conditions, nitrogen and sulfur metabolisms emerged as major pathways in the *M. intracellulare* transcriptome. Nitrogen metabolism is a crucial biological pathway that holds particular significance in the pathogenesis of mycobacteria, notably *M. tuberculosis* [54]. The adaptation of mycobacteria to host-induced stresses, such as acidic pH and nutrient deprivation within macrophages, is closely associated with nitrogen metabolism [55]. Ammonium is an important molecule in the core nitrogen metabolism of most bacteria, facilitating the biosynthesis of glutamate and glutamine, both of which are primary nitrogen donors [54]. Bacterial ammonium assimilation typically involves two pathways: a low-affinity pathway regulated by glutamate dehydrogenase (GDH) and a high-affinity pathway regulated by glutamine synthetase and glutamine oxoglutarate aminotransferase (GOGAT) [54]. In mycobacteria, the glutamine synthetase and GOGAT pathways play a significant role in ammonium assimilation, whereas the GDH pathway is primarily involved in glutamate catabolism [56, 57]. Nitrate is transported into the mycobacterial cell from the extracellular environment via the NarK2 transporter and subsequently reduced to nitrite by the nitrate reductase operon (NarGHJI) [54]. Nitrites are further converted into ammonium by the nitrite reductase enzymes NirB and NirD [54]. In our study, we observed a significant upregulation of *narGHJI* in the *M. intracellulare* transcriptome, along with increased expression of the nitrite reductase enzymes (NirB and NirD) under acidic stress conditions (depicted in Fig. 5).

Previous research has highlighted the close relationship between the nitrate reductase system and the evolutionary success of "modern" *M. tuberculosis* lineages, which exhibit enhanced virulence and infectivity compared to other *M. tuberculosis* complex species [58]. Furthermore, the multimeric nitrate reductase complex NarGHIJ expression was significantly upregulated in human lung granulomas derived from tuberculosis patients [59]. Importantly, nitrate respiration facilitated by NarGHJI results in the accumulation of nitrite, which can exert a toxic effect on bacterial cells under acidic pH conditions due to its conversion into nitric oxide, thereby exhibiting antimicrobial activity [60].

Notably, advantage of nitrate and nitrite reductase systems is their capacity to produce ammonium, serving as a buffer in acidic environments. We showed that *M. intracellulare* upregulates NirBD nitrite reductase and nitrite export proteins, such as NarK3 and NarU, under acidic stress conditions (See Fig. 4). Similar findings were observed in the hypoxic culture of *M. tuberculosis*. Akhtar et al. showed that the expression of NirBD was significantly upregulated at both the transcriptional and protein levels in a THP-1 cell-based in vitro dormancy model [61]. Furthermore, Malm et al. provided evidence that NarGHJI and NirBD in *M. tuberculosis* facilitate the assimilatory reduction of nitrate and nitrite, respectively, with GlnR as a transcriptional modulator for NirBD [62]. The upregulation of NirBD confers several advantages to mycobacteria, including the provision of ammonium for pH regulation in acidic environments and the reduction of nitric oxide toxicity. Further investigations are warranted to elucidate the role of nitrate and nitrite reductase systems in MAC, with a focus on intracellular survival mechanisms.

Previous studies suggest that sulfur metabolism and sulfur-containing metabolites play a pivotal role in the pathogenesis of mycobacteria [63, 64]. Sulfur-containing metabolites derived from mycobacteria influence bacterial infectivity and pathogenicity [65–67]. One unique major component of the cell wall, glycolipid Sulfolipid-1, induces the expression of cytokines in human tuberculosis patients [66]. Additionally, reduced sulfur-containing metabolites, such as cysteine, methionine, and coenzyme A, participate in the synthesis of essential biomolecules like proteins, lipids, and mycothiol [63]. Among these molecules, coenzyme A is a critical element in lipid metabolism, responsible for maintaining and modifying mycobacterial cell walls [68]. Also, mycothiol serves as an intracellular reducing agent that regulates cellular redox status, providing protection to bacterial cells by detoxifying electrophilic compounds, reactive oxygen and nitrogen species, as well as antibiotics [69]. In contrast, the sulfate molecule of *M. tuberculosis*, menaquinone S881, exerts a negative regulatory effect on bacterial virulence in mouse models [67]. The sulfate assimilation pathways are responsible for biosynthesis of these sulfur-containing metabolites in *M. tuberculosis*.

The bacterial sulfate assimilation pathway involves a series of enzymatic reactions responsible for the uptake and processing of inorganic sulfate from the host (see Fig. 5). In *Mycobacterium intracellulare*, this pathway initiates with the active transport of extracellular sulfate, followed by its conversion into adenosine 5′-phosphosulfate (APS) through the catalytic activity of ATP sulfurylase (as shown in Fig. 5). APS can be further phosphorylated by APS kinase, resulting in the production of 3′-phosphoadenosine 5′-phosphosulfate (PAPS), which serves as the universal sulfate donor within the bacterial cell. PAPS can be subsequently converted into sulfite by PAPS reductase and then serves as a substrate for various enzymes, including formate dehydrogenase and sulfite reductases (depicted in Fig. 5). These collective reactions constitute the sulfation branch of the sulfate assimilation pathway in *M. intracellulare*.

In the present study, we observed a significant upregulation of genes associated with sulfur metabolism. The expression of sulfate adenylyltransferase subunit 1 and 2 (OCU_15790 and 15,800) was significantly upregulated in acidic stress condition (see Fig. 4). Also, significant upregulation of PAPS reductase (OCU_18650) was observed. Furthermore, several DEGs involved in assimilatory sulfate reduction pathway including formate dyhydrogenase (OCU_20490), ferredoxin 1 (OCU_21500), and ferredoxin 2 (OCU_18660) were upregulated under acidic stress. The orthologue of sulfate adenylyltransferase subunit 1 and 2 from *M. tuberculosis* was upregulated within the macrophages and in vitro stationary phase growth [70–72]. Upregulation of the assimilatory sulfate reduction pathway indicates the accumulation of sulfide under acidic stress conditions.

Sulfide serves as a substrate for biosynthesis of cysteine and then cysteine can be converted into hydrogen sulfide (H_2_S) via various enzymes [73]. In mammalian cells, three type of H_2_S producing enzymes have been identified as follows: cystathionine β-synthase (CBS), cystathionine γ-lyase (CSE), and 3-mercaptopyruvate sulfurtransferase [73]. The presence of homologues for CBS and CSE in *M. intracellulare* genome suggest that *M. intracellulare* has the capacity to generate H_2_S. Although H_2_S, initially thought to be an intermediate metabolite of sulfur metabolism produced by bacteria, previous studies have reported that H_2_S plays important physiological roles, such as modulating the host immune response and maintaining redox homeostasis in many bacterial species [64, 74, 75]. Therefore, H_2_S can influence the intra- and extracellular environments of bacterial pathogens during infection, potentially favoring persistence of pathogen. The transcriptional response to acid stress may not be directly linked to oxidative stress. Nevertheless, when exposed to low pH, genes responsible for oxidative stress have been observed to undergo significant upregulation [76]. It is plausible that a low pH disrupts the electron transfer chain, leading to the generation of superoxide [77]. Superoxide, in turn, can initiate the formation of other reactive oxygen species, potentially inducing oxidative stress [77]. Consequently, the upregulation of assimilatory sulfate reduction pathways under acidic stress suggests the promotion of resistance to secondary oxidative stress through H_2_S production. Further studies are required to clarify the role of H_2_S in MAC pathogenesis, with a focus on modulating host immune responses and metabolism.

**Fig. 6:**
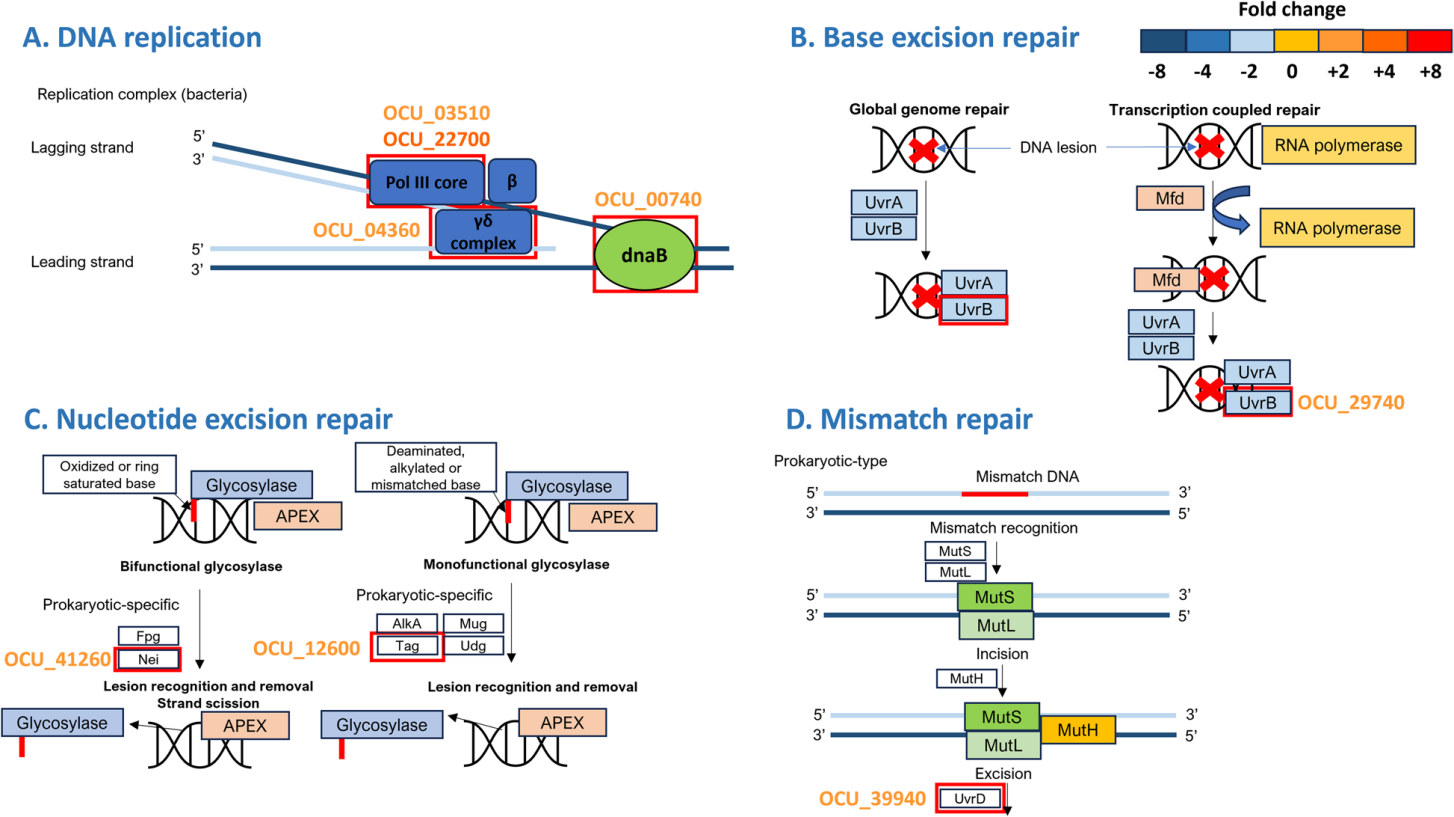
Transcriptomic pathways in *M. intracellulare* that exhibit differential expression in response to oxidative stress. Several pathways such as DNA replication **A**, Base excision repair **B**, Nucleotide excision repair **C**, and Mismatch repair **D** in *M. intracellulare* are presented here and show differential expression under acidic stress. We have depicted gene expression associated with nitrogen metabolism, with the colors of gene IDs indicating their expression levels as described in the figure. The gene expression levels of differentially expressed genes identified under acidic stress were compared with the corresponding transcripts detected under untreated control growth conditions

Under oxidative stress conditions, seven KEGG pathways were significantly activated. Among the activated pathways, all of them belonged to the genetic information processing category, except for the "tuberculosis" pathway (Fig. 4). Specifically, DNA replication associated genes such as OCU_00740 (replicative DNA helicase, *DnaB*), OCU_03510 (DNA polymerase III subunit epsilon, *dinG*), OCU_22700 (hypothetical protein), and OCU_04360 (DNA-directed DNA polymerase III subunit delta) were upregulated. Also, DNA repair associated genes, including OCU_41260 (endonuclease), OCU_12600 (DNA-3-methyladenine glycosylase I), OCU_29740 (excinuclease ABC subunit B, *uvrB*), OCU_39940 (ATP-dependent DNA helicase, *uvrD2*), OCU_39980 (Probable ATP-dependent DNA helicase), OCU_34590 (recombinase A, *recA*), OCU_04800 (DNA repair protein RadA), and OCU_33030 (holliday junction resolvase) were upregulated (Fig. 6).

Several DNA repair systems that counteract oxidative-induced mutagenesis have been described in various bacterial species, including *M. tuberculosis*. Kelley et al. revealed that DnaB helicase plays a crucial role in the transition to a dormant state in *M. smegmatis* under oxidative stress by modulating intein splicing [78]. Furthermore, DinG unwinds G4 DNA structures, which are frequently present in the mycobacterial genome and play a critical role in the regulation of mycobacterial gene expression [79]. In that regard, targeting G4 DNA structures with G4-DNA specific ligands can be a therapeutic strategy for MAC infection. UvrB and UvrC are key components of the nucleotide excision repair system in bacteria and are associated with bacterial virulence. Previous studies have shown that nucleotide excision repair genes modulate mycobacterial survival within the host. Oxidative stress results in significant upregulation of numerous DNA repair system genes such as *recA*, *dinB*, *uvrB*, *lexA*, *radA*, and *helicase* in *M. smegmatis*, suggesting their universal role for stress response in mycobacteria [80]. The expression of several *uvr* genes, including *uvrB*, was upregulated in *M. tuberculosis* within human macrophages [81]. Moreover, Darwin and Nathan provided evidence that the *M. tuberculosis uvrB* mutant exhibited a significant reduction in bacterial load within bone marrow macrophages and mouse models [82]. The recombinase A, encoded by *recA* gene contributes to *M. tuberculosis* survival by suppressing of the mitogen-activated protein kinase activity in THP-1-derived macrophages in vitro [83]. In addition, the *recA* deletion mutant of *M. bovis* BCG was more susceptible to DNA damage but showed a similar bacterial load in the BALB/c mouse model [84].

## Conclusion

In conclusion, we provide detailed insights into the transcriptomic stress response of *M. intracellulare* strain S1-36 under oxidative and acid stress conditions. Notably, exposure to acidic stress resulted in prominent changes in the transcriptome. Our results provide evidence for the importance of nitrogen and sulfur metabolism genes in the acidic stress response, including *narGHIJ*, *nirBD*, *narU*, *narK3*, *cysND*, *cysC*, *cysH*, *ferredoxin 1* and *2*, and *formate dehydrogenase*. Additionally, our results demonstrate that DNA replication and repair system genes, such as *dnaB*, *dinG*, *urvB*, *uvrD2*, *radA*, and *recA*, are indispensable for resistance to oxidative stress. Further reverse-genetics approaches, including gene silencing, targeted gene disruption, and transposon-mediated mutagenesis, are required to validate our predictions based on RNA-seq.

### Supplementary Information

**Additional file 1: Supplementary Figure 1 and 2.** Heatmaps of differentially expressed genes belong to the top 20 and 7 enriched KEGG pathways under acidic and oxidative stress conditions revealed by transcriptome profiling.

## Data Availability

All raw RNA-seq reads and processed files of transcriptome sequencing analyzed in this study are available in NCBI with the GEO accession number GSE244264 under the project number PRJNA1021764.
