# Supplementary figures and images for: Prominent transcriptomic changes in Mycobacterium intracellulare under acidic and oxidative stress
Source: BMC Genomics. 2024 Apr 17;25:376. doi: 10.1186/s12864-024-10292-4 (PMC11022373; doi:10.1186/s12864-024-10292-4)

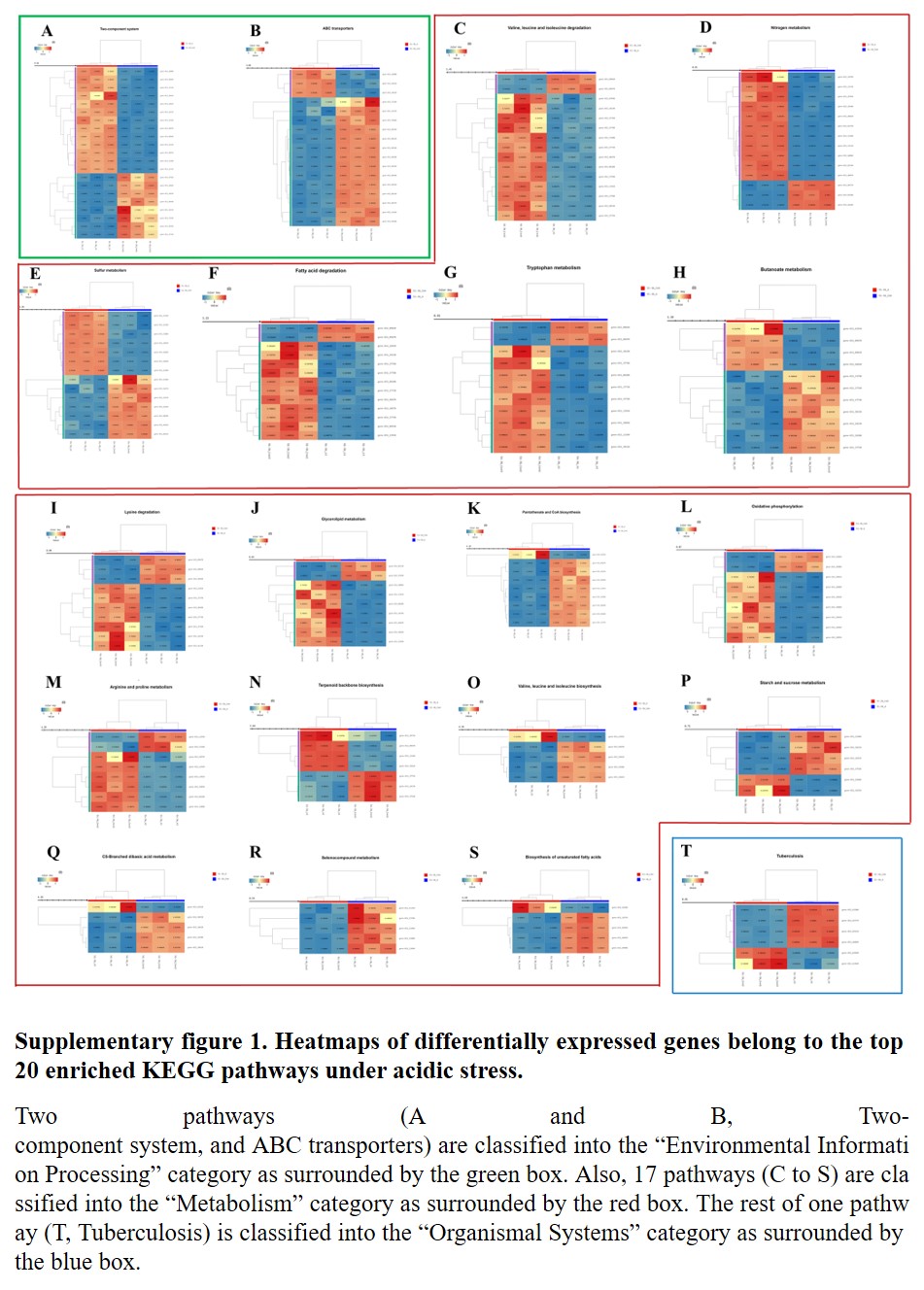

Supplement: Supplementary file 1 — Additional file 1: Supplementary Figure 1 and 2. Heatmaps of differentially expressed genes belong to the top 20 and 7 enriched KEGG pathways under acidic and oxidative stress conditions revealed by transcriptome profiling. [file 12864_2024_10292_MOESM1_ESM.zip › Supplementary Figure 1.jpg]

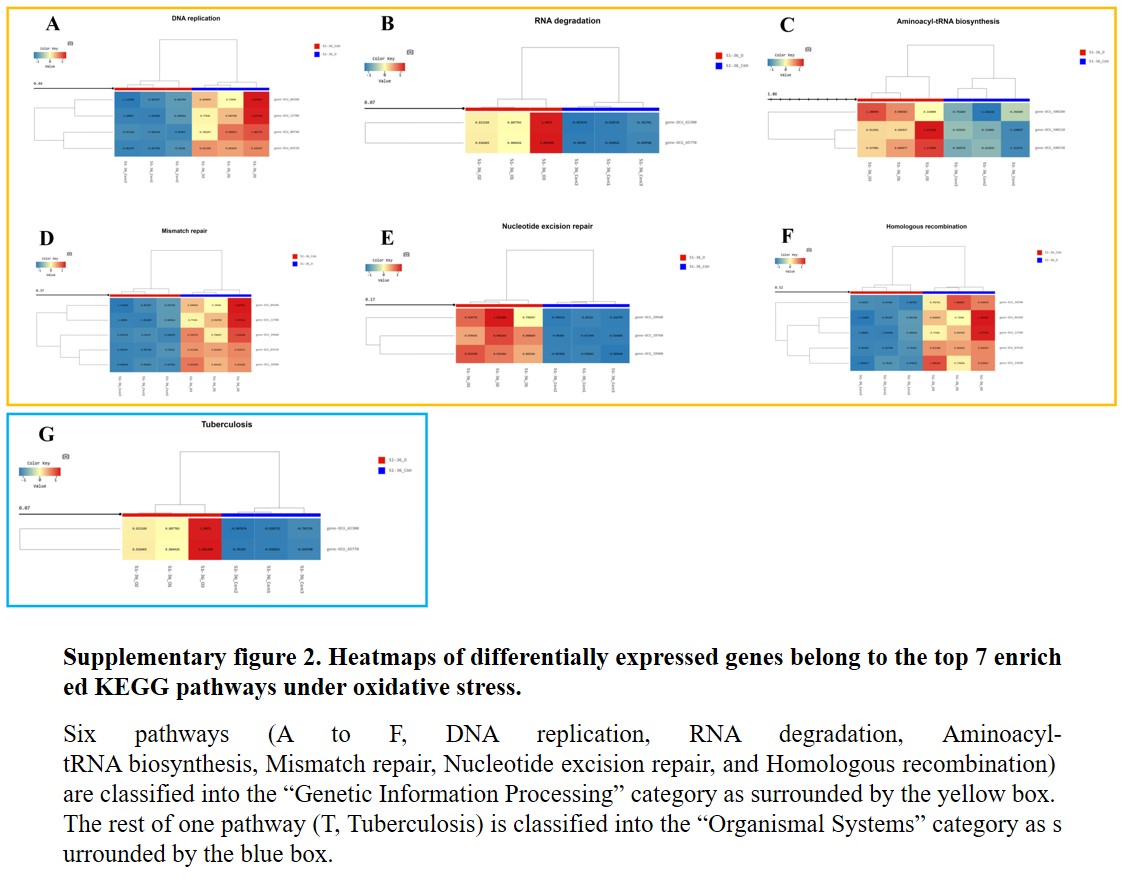

Supplement: Supplementary file 1 — Additional file 1: Supplementary Figure 1 and 2. Heatmaps of differentially expressed genes belong to the top 20 and 7 enriched KEGG pathways under acidic and oxidative stress conditions revealed by transcriptome profiling. [file 12864_2024_10292_MOESM1_ESM.zip › Supplementary Figure 2.jpg]
